# Exosomes in hepatocellular carcinoma: a new horizon

**DOI:** 10.1186/s12964-018-0315-1

**Published:** 2019-01-07

**Authors:** Rui Chen, Xin Xu, Yuquan Tao, Zijun Qian, Yongchun Yu

**Affiliations:** 1 Shanghai Municipal Hospital of Traditional Chinese Medicine, Shanghai University of Traditional Chinese Medicine, Shanghai, 200071 China; 2 Shanghai Chest Hospital, Shanghai Jiao Tong University, 241 Huaihai West Road, Shanghai, 200030 China

**Keywords:** Hepatocellular carcinoma, Exosomes, Biomarkers, Therapy

## Abstract

Exosomes are a class of extracellular vesicles released by multiple cells types including tumor cells, with a size range of 30-100 nm and a lipid bilayer membrane. Recently, the role of exosomes in cell-to-cell communication has been extensively studied, showed that exosomes can deliver their functional RNAs and proteins to recipient cells, impacting transcription and translation of recipient cells. Emerging evidence suggests that hepatocellular carcinoma (HCC) cell-derived exosomes can construct a fertile environment to support HCC cells proliferation, grow, invasion and metastasis, development of drug resistance. Circulating exosomes can be used as noninvasive biomarkers for early diagnosis, moreover as drug delivery vehicles, provide new insights into the treatment of HCC.

## Background

Liver cancer is the sixth most common cancer and the fourth leading cause of cancer-related deaths worldwide in 2018, with approximately 841,000 new cases and 782,000 deaths annually [1]. Hepatocellular carcinoma (HCC) is the most primary malignant liver tumors, and linked to hepatitis B or hepatitis C infection as well as cirrhosis [2]. Although great advancement has been achieved in diagnosis and therapeutic strategies, such as hepatic resection, liver transplantation, ablative therapy, chemoembolization, and sorafinib, the long-term survival remains daunting, owing to high rate of metastasis and relapse [3].

Exosome, a nanosized membrane vesicle, contains nucleic acids, proteins, and lipids. Surprisingly, exosomes are not only specifically target to the membrane proteins of host cells to initiate downstream signaling, but are also able to deliver genetic cargos into the cytoplasm, which provide novel mechanisms of intercellular communication. Similarly, in cancer, exosomes act as vehicles for exchange of cargos between heterogeneous populations of tumor cells and neighbor cells as well as distant cells, reprogramming tumor environment. In a review, we summarize the recent findings regarding HCC cell-derived exosomes, contribute to elucidate the molecular mechanisms underlying HCC progression and may provide a novel diagnosis and therapy strategy of HCC.

### Exosomes biogenesis

**Fig. 1:**
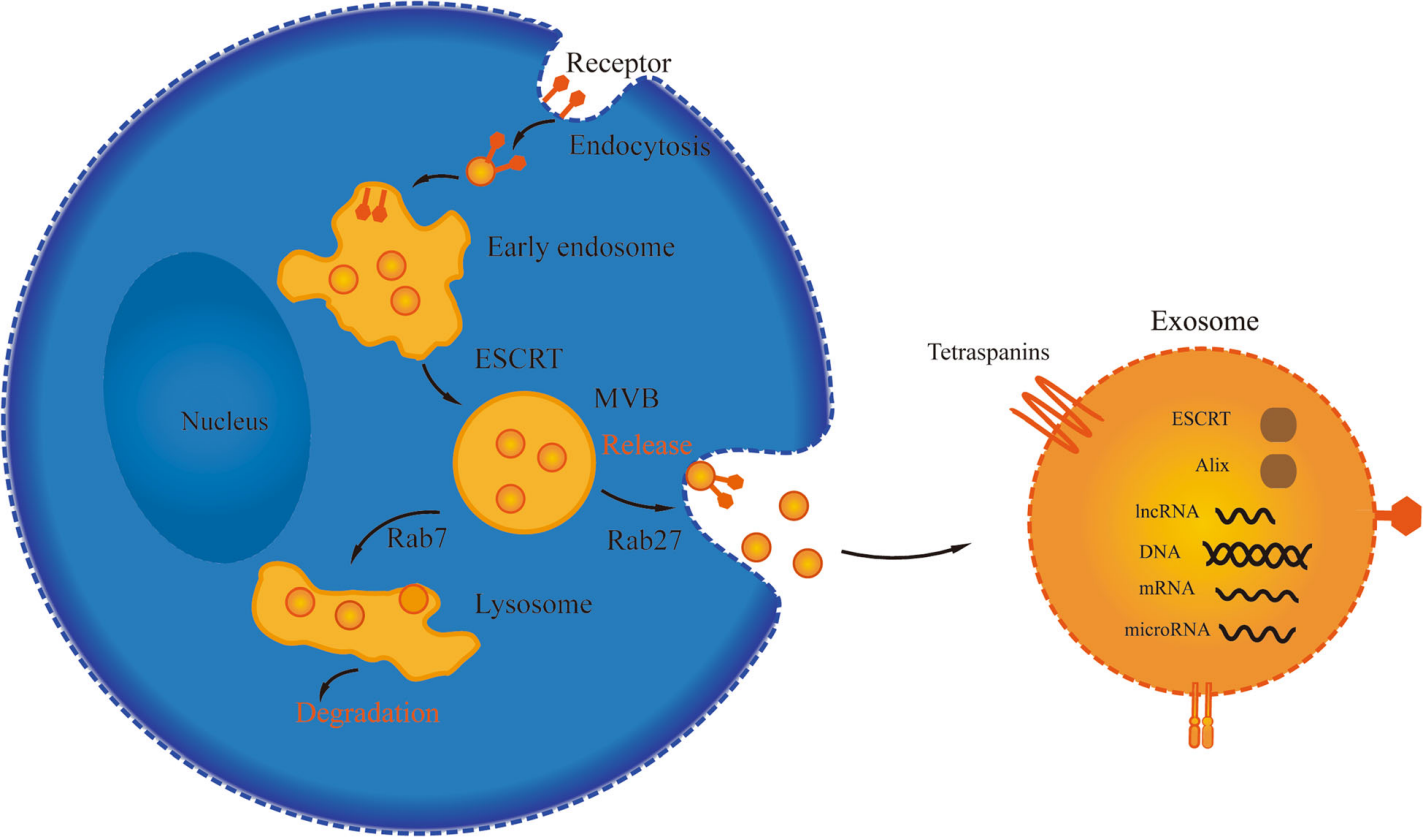
Exosomes biogenesis. Exosomes are vesicles of endocytic origin, following inward budding of the plasma membrane to form early endosomes, further inward budding of the multivesicular bodies (MVBs) generate intraluminal vesicles (ILVs), MVBs fuse with the plasma membrane, and release exosomes into the extracellular space. ESCRT and ESCRT-independent mechanism involved in exosomes biogenesis and release

Exosomes are small membrane vesicles with a size of 30–100 nm and a density of 1.13–1.19 g/ml [4–6]. Vesicles released from sheep reticulocytes during maturation were first termed exosomes by Johnstone et al. in 1987 [7]. The generation of exosomes by inward budding of the plasma membrane to form early endosomes, further inward budding of the limiting membrane inside endosome generate intraluminal vesicles (ILVs), bodies (MVBs) can either fuse with the plasma membrane, releasing of the vesicles into the extracellular space in the form of exosomes, or, alternatively, traffic to lysosomes, degrading of vesicular contents (Fig. 1) [8–11].

Endosomal sorting complexes required for transport (ESCRT), multiprotein complexes consisted of ESCRT -0, -I, -II, and -III, as well as accessory proteins (VPS4, VTA1 and ALIX), are involved in intraluminal vesicles formation and cargos sorting [12–14]. The ESCRT-0, which composes of HRS/Vps27p and STAM, is essential for initial selection of ubiquitylated cargos at the endosomal membrane, ubiquitylated cargos are first recognized by HRS, then transfer to ESCRT-I and ESCRT-II, which are responsible for membrane deformation into buds with sequestered cargos, subsequently activate ESCRT-III and the accessory proteins VPS4 ATPase, drive vesicles scission to form intraluminal vesicles. After scission, cargos are internalized in intraluminal vesicles whereas ESCRT-III remains on the outside of the membrane until it is recycled by Vps4 [15–19].

However, an ESCRT-independent mechanism involved in exosomes biogenesis and release has also been demonstrated. For example, the sphingolipid ceramide enrich in exosome, and the release of exosomes was reduced after the inhibition of neutral sphingomyelinases [20]. There are evidences showing that Rab5, Rab7, Rab27a and Rab27b of the Rab family of small GTPases participate in multivesicular endosomes trafficking and fusion with the plasma membrane or lysosome [21–23]. In addition, the tetraspanin proteins such as CD9, CD63, and CD81 have also been found to function in MVB trafficking and exosome secretion [24, 25].

### Exosomes compositon

Exosomes are released into the extracellular space by multiple cell types, including hematopoietic cells, immune cells, intestinal epithelial cells, neurons, fibroblasts, mesenchymal stem cells and tumor cells [26–28]. Owing to their endocytic origin, exosomes can fuse with membrane of recipient cells and deliver their contents into the cytoplasm [29]. Or, exosomes were internalized by recipient cells through clathrin-independent and clathrin-mediated endocytosis, such as phagocytosis, macropinocytosis and pinocytosis [30–32]. In addition, membrane proteins of the exosomes can engage the receptors of recipient cells to induce intracellular signaling [33].Therefore, the composition of exosomes are crucial determining factors in their effects.

The lipids composition of exosomes are differences with whole cell membranes, which are enriched in cholesterol, sphingomyelin, ceramide and phosphatidylserine, and generally of saturated fatty acids, while lysobisphosphatidic acid (LBPA), a lipid described in intraluminal vesicles, was not enriched [34–36].

Initial proteomic studies revealed that exosomes contain a particular subset of proteins from endosomes, the plasma membrane and the cytosol, but very little from nucleus, mitochondria, endoplasmic reticulum, and the Golgi complex [37]. Exosomes from different cell types contain membrane transport and fusion proteins (GTPases, annexins and flotillin), tetraspanins (CD63, CD9, CD81 and CD82), the ESCRT complex (TSG101, Alix), and heat shock proteins (HSP60, HSP70 and HSP90) [38–41], notably, these membrane proteins are usually used as markers for exosome identification. Except for these proteins, exosomes also include some specific proteins reflective of their parental cells, for example, integrins (αvβ5, α6β4 and α6β1) [42], MHC class I and II molecules, FasL, tumour-necrosis-factor-related apoptosis-inducing ligand (TRAIL) or programmed-cell death ligand 1(PD-L1) and prostaglandin E2 [43], as well as epidermal growth factor receptor (EGFR) [44, 45], these membrane proteins mediate interaction with specific receptors on target cells, triggering downstream signalling events.

The first identified of mRNA and microRNA in exosomes were secreted by mast cells, and in vitro translation proved that the transferred exosomal mRNA can be translated into proteins after entering recipient cell [46]. Exosomes show selectivity in their RNAs loading compared to the cells, RNAs transported by exosomes are mostly small RNA (<200 nucleotides) and fragmented mRNAs [47]. Exosomes transfer miRNAs to recipient cells, then miRNA target mRNA that regulate proteins translation involved in a wide range of biological processes [48, 49]. Apart from mRNA and miRNA, other RNAs were also identified in exosomes containing long noncoding RNA (lncRNA), transfer RNA (tRNA), ribosomal RNA (rRNA), small nucleolar RNA (snoRNA), small nuclear RNA (snRNA), small cytoplasmic RNA, silencing RNA and piwi-interacting RNA [50–53]. They are predominately fragmented mRNAs, microRNAs, and lncRNAs by exosomes transferred to recipient cells and functional in this new location, thereby impacting the transcriptome of recipient cells [54]. Noteworthy, exosomes-enclosed RNAs are protected against RNase degradation, making them ideal circulating biomarkers [55]. In additon, Balaj et al. investigated that exosomes include single-stranded DNA, containing both cDNA and genomic, as well as high levels of retrotransposons sequences [56]. There are studies indicate that exosomes also carry large fragments of double-stranded genomic DNA, encompassing all chromosomes [57, 58].

### Isolation of exosomes

The isolation of exosomes from cell culture supernatants or other bodily fluids is critical to analysis function of exosomes. Ultracentrifugation is the most widely used conventional approach for exosomes isolation [59–65]. To avoid contamination by serum exosomes, conditioned culture media was replaced by serum-free medium [64, 65], or, fetal bovine serum (FBS) previously depleted of exosomes [66–68]. In brief, exosomes were isolated by successive centrifugations at 300×g for 5 min, 2000×g for 10 min, and 10,000×g for 30 min to eliminate cells, dead cells, and cellular debris, 100,000×g for 70 min to collect exosomes fractions. The exosome pellets were washed in a large volume of PBS to eliminate contaminating proteins, and centrifuged one last time at 100,000×g for 70 min. Of note, all centrifugations were performed at 4 °C [59]. In some cases, the supernatant was filtered through a 0.22-mm filter to eliminate any remaining debris for further purification by ultracentrifugation [59, 67]. Although ultracentrifugation is most widely used gold standard for exosomes isolation, however, the method has some limitations, for example, successive centrifugations steps resulting in low yield and low recovery, as well as low purity that may contain aggregated proteins and ribonuclear protein particles [69]. Sucrose-gradient centrifugation is also commonly used isolation methods of exosomes, which extra use a sucrose cushion to eliminate more contaminants and further fractionate different vesicular density [59, 70–72]. Sucrose-gradient centrifugation is considered to isolate exosomes at a higher purity, especially greater purity and quantity mRNA profile [73, 74]. The immuno-magnetic isolation of exosomes is a versatile and rapid method for the analysis of membrane proteins of exosomes [75, 76]. However, this method is not intended for large sample volumes, and trapped exosomes may not retain full functionality even though successfully eluted from the beads surface [59]. Tauro BJ et al. discovered exosomes-specific markers abundance in LIM1863 colorectal cancer cells isolated by three different methods. They found antibody-coated magnetic beads to be the most effective methods for isolation of exosomes compare to ultracentrifugation and density gradient separation [77]. Additionally, exosomes are also isolated by using commercially available reagent, for instance, ExoQuick (System Biosciences) is the most commonly used reagent that base on polymer coprecipitation protocols [65, 66].The ExoQuick reagents precipitate exosomes by decrease the solubility, which is easy and user-friendly method, but lack specificity and may cause heterogeneous polymeric particles [78]. Caradec J et al. used two different methods to isolate exosomes form serum sample, and indicated that ExoQuick™ is an efficient and reproducible approach for exosomes quantitative studies compare to ultracentrifugation [79]. The isolation of exosomes from blood is crucial to develop exosomes as biomarkers of HCC. Plasma is a highly complex and viscous fluid with a protein concentration of 60–80 mg/ml [80]. Owing to the viscosity of plasma, it is essential to dilute plasma, and increase the speed and lengths of centrifugations [59].

However, after the isolation of exosomes, it is necessary to further identify the exosome morphology, size and marker. Exosomes exhibite typical cup-shaped morphology under transmission electron microscopy (TEM), and nanoparticle tracking analysis (NTA) the size distributions and number of exosomes, as well as western blotting identify the exosomes marker, such as TSG101,CD63 [81–83].

### Functions of exosomes in HCC

Exosomes contain specific microRNA, lncRNA and proteins that derive from the parental cells, indeed, cancer-derived exosomes can reflect the characteristics of the tumors. Most notably, exosomes can easily be obtained from a multitude of biological fluids of cancer patients, such as saliva, breast milk [84], cerebral spinal fluid [85], serum and plasma [86], urine [87], ascites [88], pleural effusions [89] and bronchoalveolar lavage fluid [90]. Furthermore, exosomes are very stable for long time storage at − 80 °C, thus, exosomes can be used as a promising biomarkers for cancer diagnosing and dynamical monitoring [4, 6, 91, 92].

Of note, tumor-derived exosomes carry a functional molecular cargo and trigger various autocrine and paracrine signaling cascades that induce malignant transformation and field cancerization. Increasing evidence showing that exosome play a significant role in tumorigenesis, growth, progression, metastasis, immune escape and drug resistance as well as treatment of cancer [4, 6, 91–95]. With the rapid development of exosomes research has helped to reveal novel mechanisms underlying HCC initiation and progression, and demonstrate that exosomes are critical intercellular messengers employed by HCC cells to architect the local and distant microenvironment.

### Exosomes as potential biomarkers for HCC

Several studies suggested that serum exosomes and their mRNA, microRNA, as well as lncRNA might serve as biomarkers for HCC screening and monitoring (Table 1).

**Table 1:** Serum exosomal-derived biomarker studies in HCC

Biomarkers (expression)	Method	Cohort (patients)	Clinical significance	References
(1) mRNA				
hn-RNPH1 mRNA↑	Taqman real-time PCR	68 HCC vs 67 LC vs 68 CHB vs 68 healthy control	Diagnostic biomarker for dividing HCC and CHB	[96]
(2) miRNA				
miR-718↓	qRT-PCR	59 HCC	Predicting biomarker for recurrence afer LT	[97]
miR-18a↑ miR-221↑ miR-222↑ miR-224↑	qRT-PCR	30 HCC vs 30 CHB vs 30 healthy controls	Diagnostic biomarker for dividing HCC and CHB	[99]
miR-101↓ miR-106b↓ miR-122↓ miR-195↓	qRT-PCR	20 HCC vs 20 CHB vs 20 LC	Diagnostic biomarker for dividing HCC and CHB	[99]
MiR-125b↑	qRT-PCR	158 HCC vs 30 CHB vs 30 LC	Predicting biomarker for recurrence and survival	[100]
miR-122↑ miR-148a↑ miR-124b↑	qPCR	5 HCC vs 5 LC	Diagnostic biomarker for dividing HCC and LC	[101]
miR-122↑ miR-148a↑ AFP↑	qPCR	5 HCC vs 5 LC	Diagnostic biomarker for dividing HCC and LC	[101]
miR-122↑	qPCR	5 HCC vs 5 LC	Diagnostic biomarker for dividing HCC and healthy	[101]
(3) lncRNA				
lncRNA-HEIH↑	qRT-PCR	35 HCC vs HCV-induced Cirrhosis vs 10 HCV-induced HCC	Diagnostic biomarker for dividing HCC and CHC	[102]
ENSG00000258332.1↑ LINC00635↑	Taqman PCR	60 HCC vs 85 LC vs 96 CHB vs 60 healthy subjects	Diagnostic biomarker for dividing HCC and CHB and LC	[103]

*CHC* chronic hepatitis C, *CHB* chronic hepatitis B, *HCV* hepatitis C virus, *LC* liver cirrhosis, *LT* liver transolantation, *qRT-PCR* quantitative reverse transcription polymerase chain reaction, *qPCR* quantitative polymerase chain reaction, *AFP* alpha-fetoprotein

*mRNA in HCC exosome* The serum exosomal heterogeneous nuclear ribonucleoprotein H1 (hnRNPH1) mRNA levels in HCC patients were remarkably higher than chronic hepatitis B patients, besides, which were associated with the portal vein tumor emboli, lymph node metastasis, Child-Pugh classification, TNM stage and overall survival [96].

*microRNA in HCC exosome* Serum exosomal miR-718 expression was significantly lower in HCC patients with larger tumour diameters and recurrence after liver transplantation [97]. The expression level of serum exosomal miR-21 was markedly higher in patients with HCC than those with chronic hepatitis B, and its expression correlated with cirrhosis and advanced tumor stage [98]. The serum exosomal miR-18a, miR-221, miR-222 and miR-224, as well as miR-125b were remarkably higher in HCC patients than chronic hepatitis B patients and liver cirrhosis patients [99, 100]. In addition, the serum exosomal miR-101, miR-106b, miR-122 and miR-195 were lower in HCC patients than chronic hepatitis B patients [99]. The levels of serum exosomal miR-122, miR-148a, and miR-124b were markedly higher in HCC than liver cirrhosis, but not different from chronic hepatitis. Furthermore, Serum exosomal miR-122, miR-148a combined with alpha-fetoprotein (AFP) were significantly distinguish early HCC from liver cirrhosis, additionally, miR-122 was the best for differentiating HCC from healthy subjects [101].

*lncRNA in HCC exosomes* Serum exosomal lncRNA-HEIH in hepatitis C virus-related HCC patients was remarkably higher than those patients with hepatitis C virus-induced cirrhosis [102]. The levels of serum exosomal lncRNAs ENSG00000258332.1 and LINC00635 in the HCC patients were significantly higher than those in liver cirrhosis, chronic hepatitis B patients and healthy subjects. A high ENSG00000258332.1 or LINC00635 level in HCC was related to lymph node metastasis, TNM stage and overall survival. In addition, a high ENSG00000258332.1 level was associated with portal vein tumor emboli. Furthermore, the combination of the 2 lncRNAs and AFP were remarkably higher sensitivity and specificity than AFP in identifying HCC from chronic hepatitis B [103].

### Exosomes and hepatocarcinogenesis

Emerging evidence suggests that HCC cell-derived exosomes mediated interaction between HCC cells and their surrounding microenvironment, educating normal cells turn into tumor cells**.** For example, HCC cell-derived exosomes delivered a functional miRNA to recipient cells, which modulated transforming growth factor β activated kinase-1(TAK1) expression and downstream signaling c-Jun NH2-terminal kinase (JNK)/p38 MAPK and nuclear factor (NF)-κB in recipient cells, thus facilitating tumorigenesis in the liver [104]. HCC cell-derived exosomes were actively internalized by adjacent adipocytes, and induced inflammatory cytokines secretion, meanwhile, activated various kinases and NF-κB signaling pathway in adipocytes, strongly supporting tumor growth and progression [105]. HCC-derived exosomes transferred their pro-tumorigenic RNAs and proteins to normal hepatocyte, which triggered PI3K/AKT and MAPK signaling pathways in host cells, moreover, increased secretion of metalloproteinases MMP-2 and MMP-9, hence facilitating tumorigenesis in normal hepatocytes [106].

### Exosomes in HCC angiogenesis

Likewise, recently reported that HCC cells-derived exosomes can transfer their biologically active lncRNAs and proteins to endothelial cells within their microenvironment, and induced the tube-like structures formation in endothelial cells, promoting angiogenesis. Cancer stem-cell-like CD90+ liver cells-derived exosomes transferred lncRNA H19 to human umbilical vein endothelial cells (HUVECs), which markedly increased the transcripts of VEGF, the most powerful pro-angiogenic cytokine, and upregulated the VEGF production and release, furthermore, induced the tube-like structures formation in endothelial cells, promoting angiogenesis [107]. In addition, vasorin, a type I transmembrane protein, was released and transferred from HCC cells to HUVECs by exosomes, and promoted angiogenesis [108].

### Exosomes and epithelial-mesenchymal transition

Epithelial-mesenchymal-transition (EMT) is a process whereby epithelial cells lose their characteristics and acquisition of the mesenchymal phenotype [109]. It is clear that EMT play a critical role in cancer progression and malignant transformation by inducing the loss of cell-cell adhesion to promote tumor cells invasion and metastasis [110].

Accumulating evidences indicated that tumor-derived exosomes carry functional molecules that activated mesenchymal-associated gene expression and induced diverse signalling in recipient cells, thereby promoting EMT and premetastatic niche formation [93, 111]. Chen et al. investigated the role of HCC cell-derived exosomes in EMT. Highly metastatic MHCC97-H cells secreted exosomes were taken up by low metastatic HLE cells, subsequently, the high expression of mesenchymal markers, such as α-SMA, N-cadherin and vimentin, as well as the low expression of epithelial marker E-cadherin were observed in HLE cells. Moreover, the levels of EMT promoters (Slug, ZEB1 and ZEB2) were increased, in contrast, the level of mesenchymal-epithelial transition (MET)-driving promoter OVOL1 was decreased in HLE cells. Further found that MAPK/ERK signalling was activated in host HLE cells, thereby undergoing epithelial-mesenchymal transition (EMT), and promoting migration, chemotaxis and invasion of the host HCC cells [112].

### Exosomes and cancer-associated fibroblasts

In tumor microenvironment, cancer-associated fibroblasts (CAFs) actively participated in the synthesis, deposition and remodelling of much of the extracellular matrix in tumor stroma, and they are regard as a source of paracrine growth factors that impact the growth of cancer cells [113], yet, in which tumor-derived exosomes may play a crosstalk role. Recently, Fang et al. showed that HCC cells-derived exosomes delivered miR-1247-3p to normal fibroblasts, miR-1247-3p directly targeted β-1,4-galactosyltransferases (B4GALT3), a protein mediating glycosylation, with the subsequent activation of β1-integrin-NF-κB signaling in fibroblasts, normal fibroblasts converted to cancer-associated fibroblasts (CAFs). Furthermore, activated CAFs secreted pro-inflammatory cytokines, such as IL-6 and IL-8, consequently promoting HCC progression and metastasis [114].

### Exosomes regulate HCC growth and progression

Of note, many studies have shown that exosomes act as vehicles for exchanged their microRNA or lncRNA between HCC cells and/or different types of cells in the tumour microenvironment, regulating HCC growth and progression. HCC cell Huh7-derived exosomes released miR-122 that was taken up by recipient HepG2 cells, interestingly, which effectively suppressed the recipient HepG2 cells growth and proliferation [115]. HCC cell-derived exosomes transferred ultraconserved lncRNA TUC339 to neighbour cells within the microenvironment, TUC339 were transcribed in host cells, promoting HCC proliferation and spread [116]. Under hypoxia, a long intergenic noncoding RNA regulator of reprogramming (linc-RoR) expression was highly increased in HCC cells, most importantly, HCC cells-derived exosomes shuttled linc-RoR between tumors cells, linc-RoR increased HCC cells viability and promoted HCC cells survival by modulated the miR-145-HIF-1a signaling [117]. HCC patients paracancer fibroblasts-derived exosomes transferred miR-320a to cancer-associated fibroblasts (CAFs) from HCC patients, in particular, miR-320a repressed its direct downstream target PBX3, simultaneously suppressed the activation of the MAPK pathway, further suppressed CAFs proliferation [118]. Liver fibroblasts-derived exosomes delivered miR-335-5p to HCC cells, which finally inhibited HCC cells proliferation [119]. Human adult liver stem cells (HLSC)-derived exosomes contain a few miRNAs with potential antitumor activity, such as miR-451, miR-223, miR-24, miR-125b, miR-31 and miR-122. Notably, HLSCs-derived exosomes transferred those miRNAs to HCC cells, which significantly inhibited growth and stimulated apoptosis of host HCC cells [120].

### Exosomes and HCC metastasis

It is well known that intrahepatic and distal metastasis is the pivotal cause of poor prognosis of HCC, whereas exosomes widely participate in this process. High motile ability of MHCC97-H cells delivered exosomes to low motile ability of MHCC97-L cells, which increased the expression of adenylyl cyclase-associated protein 1, and promoted HCC metastasis [121]. HCC cell-derived exosomes delivered miR-103 into endothelial cells, then miR-103 inhibited the expression of zonula occludens 1, VE-cadherin, and p120-catenin in endothelial cells, which attenuated endothelial junction integrity and consequently increased vascular permeability and facilitated tumor metastasis [122]. Exosomes from the serum of HCC patients transmitted lncRNA FAL1 to target Huh7 and HepG2 cells, lncRNA FAL1 competitively bound to miR-1236 of target cells, and up-regulated the ZEB1 and AFP expression, thus promoting target cells proliferation and metastasis [123].

### Function of exosomes in HCC therapies

#### Exosomes and immunotherapy of HCC

In recent years, the exosome-based cancer therapeutics has been extensively explored, revealed that the potential role of tumor-derived exosomes (TEX) and dendritic cell-derived exosomes (DEX) in cancer immunotherapy [94]. Wolfers J et al. found that TEXs transferred tumor antigens to dendritic cells, ultimately triggering potent CD8+ T cell- mediated antitumor effects on syngeneic mouse tumors [124]. The presence of MHC-I and MHC-II molecules, costimulatory molecules and other components on the surface of DEX give them the potential to promote T cells and NK (natural killer) cells mediating tumor rejection responses [125]. In addition, DEX-based phase I and II clinical trials have been carried out in advanced non-small cell lung cancer, colorectal and melanoma, suggested that DEX vaccine was feasible and well tolerated, however, the number of patients is small, thus requiring larger samples and more studies to further verify they efficiency and safety [126–129]. Recently, the role of HCC cells-derived exosome in immunotherapy has been explored. Dendritic cells (DCs) were activated by pulsed with HCC cells-derived exosomes (TEXs), particularly, TEXs carry an array of HCC antigens. TEX-pulsed DCs were taken up by HCC cells, which significantly activated T cell-dependent antitumor immunity in host HCC cells, consequently triggering markedly antitumor immune response and improving the tumor microenvironment in host HCC cell [130].

### HCC exosomes and drug resistance

HCC is highly resistant to commonly used chemotherapeutic agents, such as sorafenib, 5-fluorouracil (5-FU) and doxorubicin. Recent studies demonstrated that exosomes have an important role in drug resistance by transporting RNAs or proteins. Multidrug-resistant cell Bel/5-FU-derived exosomes delivered miR-32-5p to sensitive Bel7402 cells, and miR-32-5p suppressed its downstream target PTEN and activated the PI3K/Akt pathway in Bel7402 cells, hence inducing multidrug resistance of Bel7402 cells [131]. HCC cells delivered exosomes to the sorafenib sensitive of liver cancer cells, which activated the HGF/c-Met/Akt signaling and inhibited sorafenib-induced apoptosis of host cells, thereby enhancing sorafenib resistance in liver cancer cells [132]. HCC cells released exosomes to recipient HCC cells, linc-VLDLR, a stress-responsive lncRNA, was increased in recipient HCC cells, meanwhile, its target ATP-binding cassette, sub-family G member 2 (ABCG2) was also up-regulated, particularly reduced chemotherapy-induced cells death, leading to acquired chemoresistance in recipient cells [133].

### Exosomes as nanocarries of anticancer therapies

Due to exosomes naturally deliver nucleic acids, proteins and lipids to recipient cells, they might act as promising vectors of drugs and biological molecules. Accumulate evidences showed that exosomes as drug delivery systems has unique features, such as low immunogenicity, high biocompatibility, poorly toxic, and cross the blood-brain barrier [94, 134]. Kim MS et al. assessed the feasibility of exosome-based formulation of paclitaxel (PTX) for MDR-related anticancer therapy. They found that incorporation of PTX into exosomes significantly increased PTX cytotoxicity in drug resistant MDCKMDR1 (Pgp+) cells in vitro. Moreover, they demonstrated airway-delivered exosomes have a potent anticancer effect in lewis lung carcinoma (LLC) mouse model [135].The utility of exosomes as biological vehicles for therapeutic agents has been actively explored in HCC therapies. Adipose tissue-derived mesenchymal stem cells (AMSCs) were transfected with miR-122, and miR-122 was effectively packaged into exosomes. Furthermore, AMSCs delivered exosomes to HCC cells, interestingly, exosomal miR-122 inhibited target gene expression in host HCC cells, thereby increasing the sensitivity of HCC cells to chemotherapeutic agents, such as sorafenib, fluorouracil (5-FU) [136]. After hepatitis C virus E2 envelope glycoprotein (HCV-E2) stimulated mast cells, the level of miRNA-490 was increased in mast cells. Moreover, mast cells transferred miRNA-490 to HCC cells via exosomes, which inhibited the ERK1/2 pathway of host HCC cells, ultimately inhibiting HCC cells metastasis [137]. Propofol stimulated tumor-associated macrophages to secrete exosomes, more importantly, exosomes were taken up by HCC cells, indeed, miR-142-3p expression was increased and its target RAC1 was significantly down-regulated in host HCC cells, resulting in inhibition of HCC cells growth [138].

## Conclusion

**Fig. 2:**
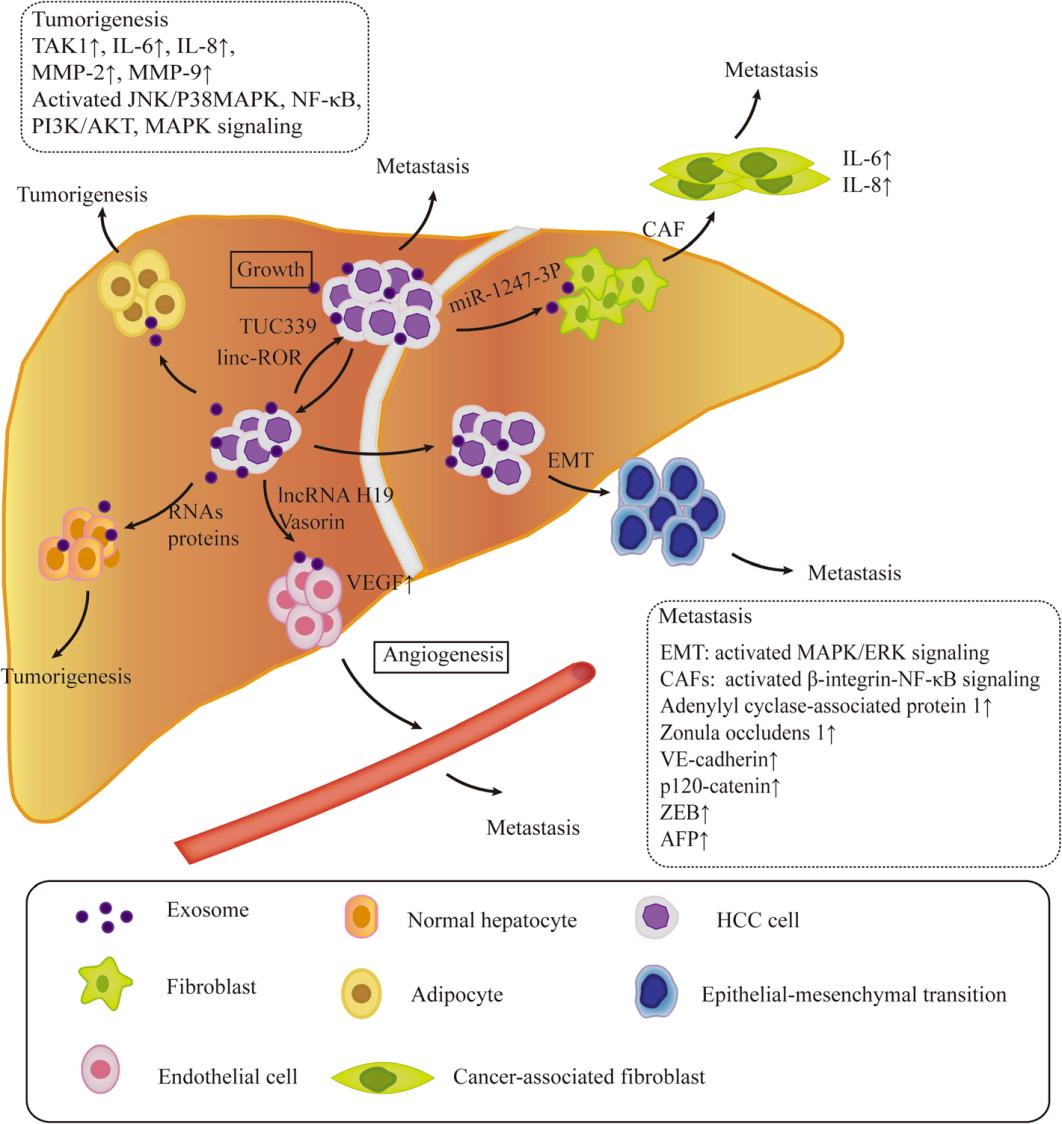
Functions of exosomes in HCC. Exosomes play a significant role in mediating interaction between HCC cells and their surrounding microenvironment. HCC cells-derived exosomes transferred their biologically active RANs and proteins to recipient cells, and triggered various signaling in recipient cells, facilitating tumorigenesis, angiogenesis, HCC cells growth and metastasis

Indeed, exosomes play an improtant role in HCC cells communication with their microenvironment, and provide fertile soil for the seed, thereby facilitating HCC proliferation and metastasis (Table 2, Fig. 2). Nonetheless, there are many problems remain to be elucidated. How miRNA, lncRNA and proteins are sorted to exosomes, and whether or not exosomes uptake is a cell type specific process need to intensive researches. In experimental research, successfully extracting exosomes from cell culture supernatant is challenging and expensive, which limit the study of exosomes. Importantly, the serum of cancer patients contain plenty of exosomes [139], which is beneficial for investigating exosomes as biomarkers for cancers screening and monitoring. Large sample studies are needed to select those exosomal RNAs and proteins with high specificity and sensitivity. Whether exosomes can regulate adaptive immunity in HCC cells microenvironment need further researches, which may offers potential therapeutic strategies for HCC. In addition, new therapeutic agents can be developed utilizing exosomes as biological vehicles.

**Table 2:** Exosomal cargos detected in HCC and their target and clinical relevance

Exosomal cargos	Target	Biological/clinical relevance	Reference
(1) miRNA			
miRNA	TAK1	Facilitated tumorigenesis	[104]
miR-1247-3p	B4GALT3	Converted normal fibroblasts to cancer-associated fibroblasts (CAFs)	[114]
miR-122		Suppressed HCC cells growth and proliferation	[115]
miR-320a	PBX3	Suppressed CAFs proliferation	[118]
miR-335-5p		Inhibited HCC cells proliferation	[119]
miR451,miR223, miR24,miR125b miR31,and miR122		Inhibited HCC cells growth and stimulated apoptosis	[120]
miR-103		Facilitated tumor metastasis	[122]
miR-32-5p	PTEN	Induced multidrug resistance in Bel7402 cells	[131]
(2) lncRNA			
lncRNA H19		Promoted angiogenesis	[109]
TUC339		Promoted HCC proliferation and spread	[116]
linc-RoR	miR-145	Increased HCC cells viability and promoted HCC cells survival	[117]
lncRNA FAL1	miR-1236	Promoted Huh7 and HepG2 cells proliferation and metastasis	[123]
linc-VLDLR	ABCG2	Leaded to acquired chemoresistance in HCC cells	[133]
(3) Proteins			
proteins		Facilitated tumorigenesis in normal hepatocytes	[106]
Vasorin		Promoted angiogenesis	[108]

TAK1 transforming growth factorβactivated kinase-1, B4GALT3 β-1,4-galactosyltransferases, PTEN phosphatase and tensin homolog, PBX3 pre-B-cell leukemia transcription factor 3, ABCG2 ATP-binding cassette, sub-family G member 2

## Abbreviations

5-FU: 5-fluorouracil
ABCG2: ATP-binding cassette, sub-family G member 2
AFP: Alpha-fetoprotein
AMSCs: Adipose tissue-derived mesenchymal stem cells
B4GALT3: β-1,4-galactosyltransferases
CAFs: Cancer-associated fibroblasts
CHB: Chronic hepatitis B
DCs: Dendritic cells
EGFR: Epidermal growth factor receptor
EMT: Epithelial-mesenchymal transition
ESCRT: Endosomal sorting complexes required for transport
FBS: Fetal bovine serum
HCC: Hepatocellular carcinoma
HCV-E2: epatitis C virus E2 envelope glycoprotein
HLSC: Human adult liver stem cells
hnRNPH1: Heterogeneous nuclear ribonucleoprotein H1
HUVECs: Human umbilical vein endothelial cells
ILVs: Intraluminal vesicles
LBPA: Lysobisphosphatidic acid
linc-RoR: Long intergenic noncoding RNA regulator of reprogramming
lncRNAs: Long noncoding RNAs
MVB: Multivesicular body
NF: Nuclear factor
NTA: Nanoparticle tracking analysis
PD-L1: Programmed-cell death ligand 1
rRNA: Ribosomal RNA
snoRNA: Small nucleolar RNA
snRNA: Small nuclear RNA
TAK1: Transforming growth factor β activated kinase-1
TEM: Transmission electron microscopy
TRAIL: Tumour-necrosis-factor-related apoptosis-inducing ligand
tRNAs: Transfer RNAs
